# A Dual-Model Framework with Gramian Angular Field and Spatio-Temporal Attention for Rapid Gas Identification and Concentration Prediction

**DOI:** 10.3390/s26102953

**Published:** 2026-05-08

**Authors:** Wenyan He, Wen Xin, Qingfeng Wang

**Affiliations:** State Key Laboratory of Integrated Optoelectronics, College of Electronic Science and Engineering, Jilin University, Changchun 130012, China; hewy24@mails.jlu.edu.cn (W.H.); xinwen25@mails.jlu.edu.cn (W.X.)

**Keywords:** electronic nose, gas identification, concentration prediction, Gramian angular field, recurrent neural network, attention mechanism

## Abstract

Rapid and accurate gas identification and concentration prediction are of critical importance for industrial safety, medical diagnostics, and environmental monitoring. However, signal distortion in complex environments and feature loss during data processing often degrade prediction accuracy and response speed. To address these challenges, this study proposes a dual-model framework for electronic nose systems. A gas classification model transforms time-series sensor data into two-dimensional feature maps using a composite Gramian Angular Field representation and end-to-end classification using a convolutional neural network (CNN). A gas concentration prediction model integrates a multi-branch attention mechanism, a CNN, and a bidirectional gated recurrent unit to capture spatial–temporal dependencies. A cascaded identification–prediction scheme is further developed to mitigate data distribution heterogeneity and enhance model robustness. The proposed method supports both single-label and multi-label tasks and exhibits strong adaptability under complex conditions, including low concentrations, varying humidity, and gas mixtures. Validation on public and laboratory-collected datasets demonstrates that, using only initial response-stage data, the classification model achieves 100% identification accuracy, while the prediction model attains R^2^ > 0.99 for the majority of target gases. These results confirm that the proposed framework provides an efficient and robust solution for rapid qualitative identification and quantitative prediction in electronic nose systems.

## 1. Introduction

Gas analysis is increasingly important in environmental monitoring [1], industrial safety [2], disease diagnosis [3,4], and food quality control [5]. For example, real-time monitoring of combustible gases is essential for preventing industrial accidents [6], while the prediction of atmospheric pollutants provides scientific support for environmental governance and policy-making [7,8,9]. In the medical field, breath gas analysis has also been utilized to evaluate physiological conditions and assist clinical diagnosis.

Conventional gas detection techniques, such as gas chromatography–mass spectrometry (GC–MS), achieve accurate qualitative and quantitative analysis through ion–molecule reactions induced by chemical ionization. However, their performance depends heavily on sample preparation and experimental conditions [10], and they suffer from long detection cycles, complex operation, and high cost, which limit their applicability in real-time and portable scenarios [11]. In contrast, metal oxide semiconductor (MOS) gas sensors have gained increasing attention due to their low cost, simple sensing mechanism, and ease of integration. To overcome the limitations of individual sensors, such as poor selectivity and sensitivity to environmental variations (e.g., temperature and humidity), sensor array-based systems have been developed [12,13]. Inspired by biological olfactory mechanisms, these systems (commonly referred to as electronic noses) consist of sensor arrays, signal acquisition modules, and pattern recognition algorithms [14,15], among which pattern recognition algorithms play a critical role in overall system performance.

Early efforts in gas identification and concentration prediction mainly used statistical analysis and conventional machine learning methods. Principal component analysis (PCA) reduces data dimensionality by preserving the dominant variance [16], while linear discriminant analysis (LDA) enhances class separability by maximizing the ratio of between-class to within-class scatter [17]. K-nearest neighbor (KNN) and Support Vector Machine (SVM) have been widely used for gas classification [18,19]. Multiple linear regression (MLR), partial least squares regression (PLSR), and support vector regression (SVR) are commonly employed for concentration estimation [20]. Nevertheless, these traditional approaches typically rely on handcrafted features, such as steady-state responses, peak values, and response areas, which inadequately capture the nonlinear and temporal dynamics of sensor responses, thereby limiting model performance.

Recent advances in deep learning have significantly enhanced gas sensing performance. Convolutional neural networks (CNNs), leveraging their end-to-end feature learning capability, have been successfully applied to one-dimensional, two-dimensional, and three-dimensional data representations [21,22]. For example, a small-scale CNN model proposed in [23] achieves effective gas classification without complex preprocessing. Similarly, the method in [24] integrates the discrete wavelet transform (DWT) with CNN to construct a dynamic wavelet CNN, yielding high classification accuracy. However, these approaches typically rely on predefined signal transformation techniques and mainly focus on gas classification, with limited attention to concentration prediction. Furthermore, most existing studies focus on single-component gas identification or qualitative analysis of multi-component mixtures [25,26,27], whereas high-precision quantitative prediction remains insufficiently explored [28].

To model the temporal dynamics of sensor responses, recurrent neural networks (RNNs) and their variants have been widely used for gas concentration estimation. Transformer-based architectures with attention mechanisms have demonstrated superior performance in sequence modeling [29,30,31]. Hybrid CNN–RNN frameworks have been further proposed to jointly perform classification and regression. For instance, the method in [32] employs WCCNN-BiLSTM for gas classification followed by GRU for concentration prediction, while [24] introduces a many-to-many LSTM structure to improve prediction accuracy. Despite these advances, several challenges remain. First, most existing approaches require full-length sensor response sequences, resulting in high computational cost and slow recognition speed. Second, separate models are typically designed for classification and regression tasks, increasing system complexity and preprocessing overhead. Third, data distribution discrepancies caused by heterogeneous sources can degrade model generalization for single-gas scenarios.

To address these limitations, this study proposes a unified deep learning framework for gas identification and concentration prediction that efficiently uses dynamic response information. Specifically, only the critical segment of the sensor response, from the initial reaction to the steady-state phase, is used for modeling, which reduces data redundancy while preserving discriminative features. Furthermore, to mitigate the impact of data distribution heterogeneity in single-gas scenarios, we develop a cascaded identification–prediction system that enhances robustness and generalization. The main contributions of this work are summarized as follows:

(1) A composite Gramian Angular Field (GAF) representation that fuses summation and difference modes is proposed to transform time-series sensor data into two-dimensional feature maps, capturing both covariance structures and dynamic variations. A CNN-based model is then employed for end-to-end gas classification.
(2) A feature extraction framework with positional encoding and multi-branch attention mechanisms is designed. A channel-wise bidirectional gated recurrent unit (Bi-GRU) is further incorporated to capture spatial–temporal dependencies, enhancing concentration prediction accuracy.
(3) A cascaded identification–prediction system is developed for single-gas sensing scenarios, which mitigates data distribution heterogeneity and enhances model robustness.
(4) The proposed method supports both single-label and multi-label classification, and demonstrates strong adaptability under complex conditions, including low concentrations, varying humidity and mixed-gas concentration prediction.

## 2. Datasets and Preprocessing

### 2.1. Datasets

This section describes four datasets for model validation, including three publicly available datasets and one collected using our designed electronic nose system. These datasets are selected to establish a comprehensive validation pipeline, covering multicomponent gas mixtures, low-concentration and humidity-interference scenarios, cross-device variability, and real-world laboratory deployment, thereby ensuring the effectiveness and generalizability of the proposed method.

#### 2.1.1. Dataset I

Dataset I consists of time-series signals from the UCI Machine Learning Repository, collected by Fonollosa, J. et al. [33]. In this study, it is used to evaluate model performance for both gas classification and concentration prediction. The data acquisition platform integrated eight metal oxide (MOX) gas sensors with temperature and humidity monitoring modules in a controlled wind tunnel environment for dynamic gas mixture measurements. Two independently controlled gas sources were employed: Source 1 released ethylene (C_2_H_4_), while Source 2 delivered either methane (CH_4_) or carbon monoxide (CO). Binary gas mixtures (C_2_H_4_/CH_4_ or C_2_H_4_/CO) were generated through precise flow ratio adjustments. Each gas component was regulated at four concentration levels (zero, low, medium, and high); the details are provided in Table 1. A total of 30 distinct mixtures (15 C_2_H_4_/CH_4_ and 15 C_2_H_4_/CO combinations) were constructed, with each concentration ratio replicated six times, yielding 180 samples. Each sampling trial lasted 300 s, comprising an initial 60 s without gas inflow, followed by 180 s of exposure to a predefined gas mixture, and a final 60 s for gradual gas discharge. The sensor responses were sampled at 50 Hz.

#### 2.1.2. Dataset II

Dataset II (publicly available on UCI) is from the Javier Burgués group [34]. We use it to evaluate concentration prediction models under low-concentration conditions across varying humidity levels. Dynamic gas mixtures were generated inside a 250 cm^3^ test chamber by blending three gas streams under the control of mass flow controllers (EL-FLOW Select, Bronkhorst): carbon monoxide (CO, 1600 ppm in synthetic air), humid synthetic air, and dry synthetic air. An SHT75 sensor from Sensirion logged the internal temperature and relative humidity every 5 s. This dataset contains 1300 samples measured at 10 concentration levels (0–20 ppm) with randomized relative humidity (15–75%). Each measurement cycle consisted of a 15-min cleaning with synthetic air followed by a 15-min CO exposure, both at a constant flow rate of 240 mL/min. The sensing unit was an array of 14 temperature-modulated MOX gas sensors commercially available from Figaro Engineering (7 units of TGS 3870-A04) and FIS (7 units of SB-500-12). The operating temperature of each sensor was cyclically modulated via a built-in heater following a repeated voltage sequence: 0.9 V for 5 s, 0.2 V for 20 s, 0.9 V for 5 s, and 0.2 V for 25 s. The sensor output voltage was sampled at 50 Hz.

#### 2.1.3. Dataset III

Dataset III was also obtained from the UCI Repository, provided by J. Fonollosa et al. [35]. We use it to evaluate the performance of the proposed identification–prediction system under inter-device variability and sensor drift. The experimental setup consisted of five identical detection units. Each unit contained an array of eight MOX gas sensors, with individually controlled heater voltages, housed in a 60 mL chamber. The four target gases, ethylene, ethanol, carbon monoxide, and methane, were supplied from standard gas cylinders. Each gas was blended with synthetic air using MFCs to produce a single-component gas stream across ten different concentration levels, as listed in Table 2. The experimental procedure for each measurement was as follows: clean air was introduced for 50 s, followed by target gas exposure for 100 s, and finally clean air purging for 450 s to recover the sensor. Sensor response signals were acquired continuously over 22 days at a sampling frequency of 100 Hz, yielding a total of 640 time-series samples.

#### 2.1.4. Dataset IV

**Figure 1:**
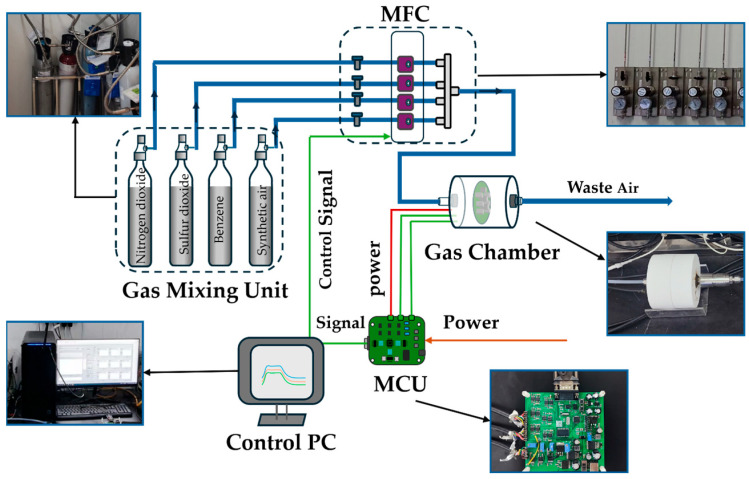
The designed gas sensing experimental setup in our lab.

To evaluate the proposed identification–prediction multi-gas prediction, we developed an E-nose experimental device and collected a real-time dataset as Dataset IV. The experimental setup, shown in Figure 1, is composed of a sensor array, a gas path system, and a signal acquisition unit.

The gas sensor array consists of nine MOX sensors, including three WSP1110, three WSP2110 (from Winsen Electronics Technology Co., Ltd., Zhengzhou, China), and three MQ136 sensors (from China). This array was designed to detect three target gases: nitrogen dioxide (NO_2_), benzene (C_6_H_6_), and sulfur dioxide (SO_2_). To obtain diverse response characteristics, the three sensors of each type operated at different heating voltages: 4.5 V, 5.0 V, and 5.5 V. Chamber humidity and temperature were monitored in real time using a DHT11 sensor and a DS18B20 sensor (both from China), respectively. The gas delivery system comprises gas cylinders, MFCs, a gas chamber, and an exhaust duct. The cylinders supply the target gases, while the MFCs precisely regulate the ratio of target gas to air, enabling controlled concentration levels and maintaining continuous gas flow through the chamber. Each experimental procedure follows a cyclic sequence of air purging, target gas exposure, and air purging. Each experiment lasts 660 s, comprising a 360 s gas exposure phase followed by a 300 s air cleaning phase, with a sampling frequency of 15 Hz. The resulting dataset covers 20 concentration gradients for each of the three gases. Detailed information is provided in Table 3.

The signal acquisition module centers on an STM32F103C8T6 microcontroller (manufactured by STMicroelectronics, a Swiss company based in Geneva, Switzerland) and includes a signal conditioning circuit with voltage division, RC filtering, and a voltage follower. Analog-to-digital conversion is performed using a built-in 12-bit ADC (integrated within the chip), and data are transmitted to the upper computer via a serial interface. The software adopts a coordinated upper–lower computer architecture. The lower computer runs the FreeRTOS real-time operating system to manage parallel tasks, including sensor data acquisition and conversion, temperature and humidity reading, OLED display, and serial communication. The upper computer, developed in LabVIEW, provides a graphical user interface for serial port configuration, real-time waveform display, data storage, and interactive control.

**Figure 2:**
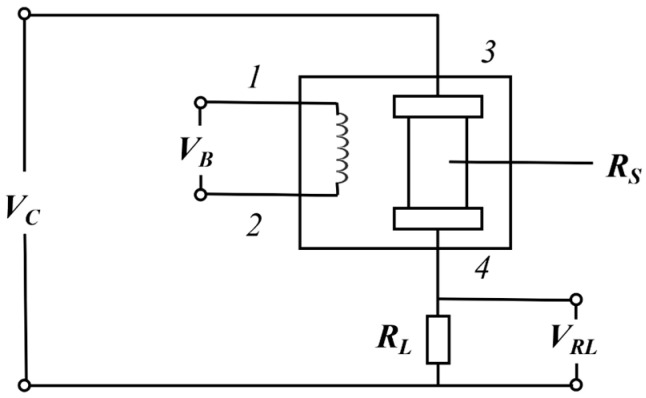
Sensor testing circuit.

Figure 2 shows the test circuit of the sensors. The heating voltage (*V_B_*) is applied to the integrated heater within the sensor, allowing the gas-sensitive element to fully react with the target gas at a specific temperature, thereby ensuring consistency of the output detection signal. The operating voltage *V_C_* is applied across the series circuit consisting of the sensor and the load resistor, converting changes in the sensor resistance into voltage variations. The primary output signal is given by $V_{RL}=R_{L}V_{C}/(R_{L}+R_{S})$, where *R_S_* is the sensor resistance and *Vc* is the applied operating voltage.

**Figure 3:**
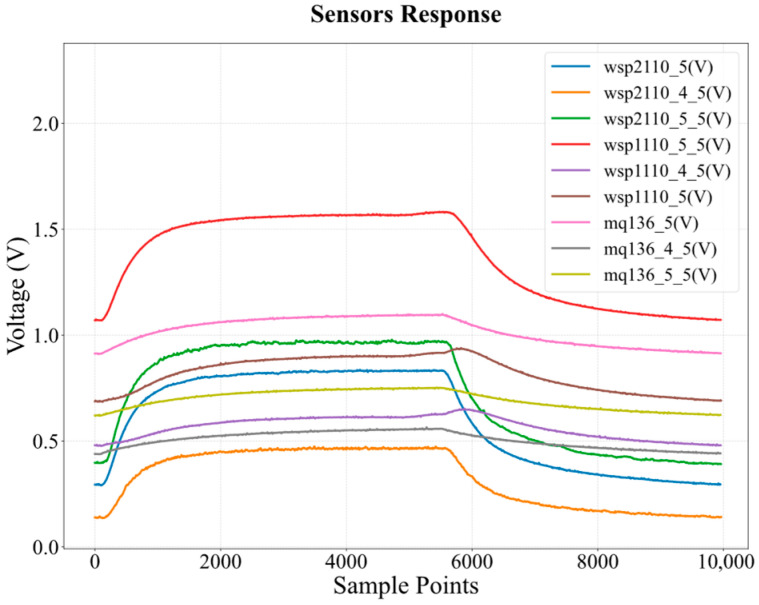
The response curves of the nine-sensor array when exposed to 7.5 ppm C_6_H_6_.

Figure 3 shows the actual response curves of the nine-sensor array when exposed to 7.5 ppm C_6_H_6_, where all sensors exhibit measurable resistance changes. Since C_6_H_6_ is a reducing gas, it reacts with the oxygen species adsorbed on the sensor surface, leading to a decrease in the sensor resistance (*R_s_*) and, consequently, an increase in the voltage division (*V_RL_*) across the load resistor. This voltage variation directly records the kinetics of the chemical adsorption–desorption processes occurring on the sensor surface. Data acquisition at 15 Hz yielded 5400 and 4500 points per sensor during the 360 s exposure and 300 s cleaning phases, respectively. Each experimental sample thus contains 89,100 points across nine sensors, capturing rich temporal detail across the full response–recovery cycle.

### 2.2. Data Preprocessing

To address challenges associated with high-dimensional data, environmental noise, and redundancy in raw sensor-array time-series data, we apply a preprocessing procedure that includes data segment extraction, exponentially weighted moving average (EWMA) smoothing, down-sampling, and data augmentation.

First, segments spanning from gas exposure onset to the stabilization of sensor response are extracted. This phase contains transient characteristics with discriminative information for gas identification and quantification. Using only this phase enables rapid analytics because it avoids the latency of full steady-state monitoring and reduces data dimensionality for efficient computation.

**Figure 4:**
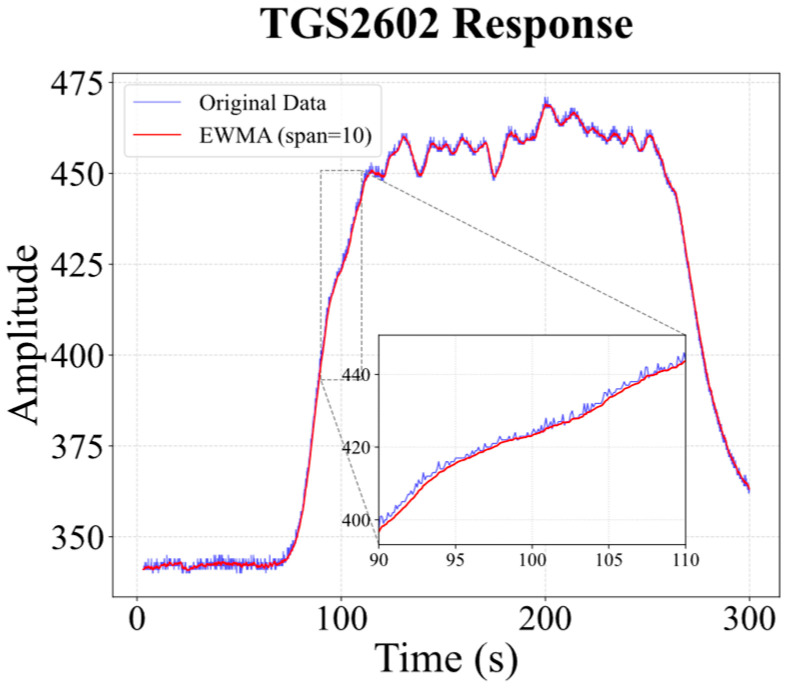
Comparison of raw and EWMA-smoothed (span = 10) signals for the TGS2602 sensor in Dataset I, featuring the global response profile and a magnified view of the transient phase.

Subsequently, EWMA smoothing is applied to suppress environmental and circuit noise. The EWMA output *x_s,t_* at time *t* for sensor *s* is calculated recursively as

(1) $$x_{s,t}=\alpha \cdot x_{s,t}^{o}+(1-\alpha )\cdot x_{s,t-1}$$

where $x_{s,t}^{o}$ is the raw observation at time *t* for sensor *s*, and $x_{s,t-1}$ is the smoothed value at the previous time step. The smoothing factor *α* is determined by a span parameter, which is set to 10 in this study, i.e., $\alpha =2/\left(10+1\right)$. Figure 4 illustrates the smoothing effect of EWMA (span = 10) on a representative sensor response. Compared with the raw signal (blue), the processed curve (red) suppresses random fluctuations and spikes, clarifying the overall trend across baseline, response, and recovery. A magnified view of the transient phase confirms that key dynamic features, onset timing and slope are well preserved, with no noticeable delay or distortion. EWMA thus improves signal quality while retaining the transient information relevant for gas concentration estimation.

Finally, down-sampling combined with sliding-window-based data augmentation was applied to reduce data dimensionality and address the limited sample size of the dataset. Consider a raw sequence from a single sensor sampled at 100 Hz comprising 1000 time points, denoted as $X_{S}=[x_{s,1},x_{s,2},\ldots ,x_{s,1000}]$, where $x_{s,i}$ represents the response value of the *s*-th sensor at the *i*-th sampling instant. Down-sampling was performed by extracting one data point every ten points, forming a segment that yields a data matrix of dimension 100 × *S*, which constitutes one data sample. Each original concentration sample was further divided into 10 such samples via a sliding window approach, generating augmented instances $\left[x_{s,1},x_{s,11},\ldots ,x_{s,991}\right]$, $\left[x_{s,2},x_{s,12},\ldots ,x_{s,992}\right]$, $\left[x_{s,3},x_{s,13},\ldots ,x_{s,993}\right]$, …, $\left[x_{s,10},x_{s,11},\ldots ,x_{s,1000}\right]$. This method effectively expands the sample size tenfold while preserving the key dynamic characteristics of the data. As Dataset II already contains a sufficient number of samples, no further augmentation was performed. After applying the above procedures, the resulting sample sizes for Datasets I–IV are 1800, 1300, 6400, and 1200, respectively.

The label encoding strategy for the classification task is as follows: For pure, single gases, a single-label encoding scheme is employed, wherein each gas category is represented by a distinct integer (i.e., 0, 1, 2, …), as illustrated in the Label column of Table 2 and Table 3. For gas mixtures, a multi-label encoding scheme is adopted, where the presence of a specific gas is encoded as 1 and its absence as 0, as shown in Table 4.

## 3. Method

**Figure 5:**
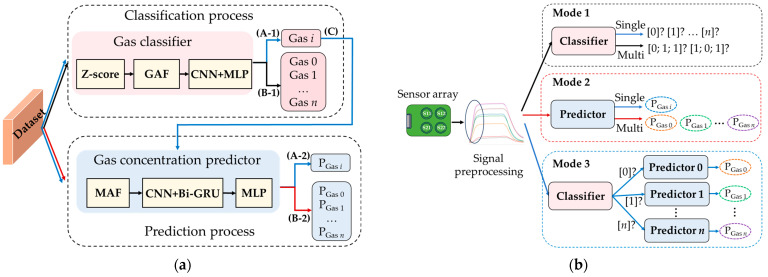
Framework of the proposed method. (**a**) Gas classification and concentration prediction process; (**b**) dynamic activation of functional branches based on task objectives.

To address the diverse requirements of gas detection in practical applications, this paper proposes a novel neural network architecture, as illustrated in Figure 5a. The framework comprises two models: a gas classifier and a gas concentration predictor. The classifier integrates a Gramian Angular Field transformation module and a multi-branch convolutional neural network module. The predictor consists of a multi-modal attention fusion (MAF) module, a CNN module, and a Bi-GRU module. Both the classifier and the predictor are equipped with an MLP module for output dimensional transformation. These components collectively enhance gas identification accuracy and concentration prediction performance, forming a versatile and robust detection system adaptable to diverse tasks in complex environments. Leveraging preprocessed sensor array data, the system dynamically activates corresponding functional branches according to the task objective, as illustrated in Figure 5b. Specifically:

(1) Gas species identification: For tasks requiring only the discrimination of gas types, the preprocessed data are fed into a classifier (data flow corresponds to paths (A-1) and (B-1) in Figure 5a). This classifier supports both single-class and multi-class modes, enabling accurate identification of all gas components present in a sample.
(2) Concentration prediction for mixed and single gases: The preprocessed data are fed into a predictor (data flow corresponds to paths (A-2) and (B-2) in Figure 5a). The number of output units equals the number of gas species, with each unit responsible for estimating the concentration of a specific gas. The proposed model can achieve high prediction accuracy even at low concentrations.
(3) Single-gas identification and concentration prediction: In scenarios where only a single gas is known to be present but both its type and concentration need to be determined, the system employs a classifier–predictor cascade architecture (shown by the blue line path ((A-1)–(C)–(A-2)) in Figure 5a). In this architecture, a front-end classifier identifies the gas type and routes the data to a dedicated regression sub-network specific to that gas category for concentration prediction. This gas-specific design mitigates interference caused by differing gas data distributions in a unified model, thereby improving prediction accuracy and robustness.

### 3.1. Gas Classifier

**Figure 6:**
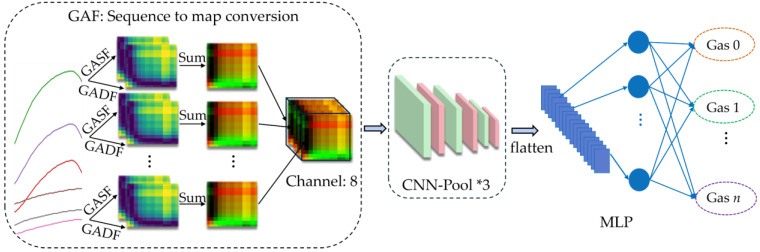
Gas classifier framework.

As illustrated in Figure 6, the proposed gas classifier framework comprises three main modules: a GAF transformer that converts raw time-series signals from the sensor array into global image representations; a multi-stage CNN that serves as a hierarchical feature extractor for multi-scale and multi-dimensional representations; and an MLP output module that maps high-dimensional features to the target classification space. This architecture integrates time-series image encoding with deep convolutional feature learning to enhance classification robustness.

First, raw time-series signals from each sensor are independently transformed using the Gramian Angular Summation Field (GASF) and the Gramian Angular Difference Field (GADF). This process converts one-dimensional temporal sequences into two-dimensional images with explicit spatial structure, enabling temporal dependencies and dynamic variations to be represented visually. Second, the two images are fused into a unified composite image. By integrating GASF’s sensitivity to global signal trends with GADF’s ability to encode temporal differences, the fused representation achieves stronger descriptiveness and discriminability. Finally, the composite images from all sensors are concatenated along the channel dimension and fed into a CNN, which performs multi-scale feature learning to capture nonlinear interactions and cross-sensor dependencies. The MLP then maps the extracted embeddings to the classification space, enabling precise identification of both individual gas species and gas mixtures.

#### 3.1.1. GAF

To improve sensor time-series feature representation, the Gramian Angular Field method is introduced. It preserves temporal dependencies via polar coordinate mapping and uses trigonometric functions to quantify correlations between data points, forming a Gramian matrix that encodes dynamic 1D time series as 2D image features [36]. GAF comprises GASF and GADF. Let $X_{s}=\left(x_{s,1},x_{s,2},\cdots ,x_{s,n}\right)$ denote the 1D sample data of the *s*-th sensor, where *n* is the number of sampling points, and s=1,2,⋯,S, with *S* being the total number of sensors in the array. First, $x_{s,i}$$\left(i=1,2,\cdots ,n\right)$ is transformed into the normalized value $x_{s,i}^{’}\in [0,1]$ using the following formula:

(2) $$x_{s,i}^{’}=\frac{x_{s,i}-\min\left(X_{S}\right)}{\max\left(X_{S}\right)-\min\left(X_{S}\right)}$$

Then, for each sensor *s*, the normalized time series $X_{s}\in {\mathbb{R}}^{n}$ is mapped to polar coordinates using angle encoding $\theta_{s,i}=\arccos(x_{s,i}^{’})$ and radius encoding $r_{s,i}=i/n$, and *i* represents the *i*-th sampling number. Next, the reconstructed sequence in polar coordinates is transformed into two types of 2D feature images (${\mathrm{GASF}}_{s}$ and ${\mathrm{GADF}}_{s}$) through GASF and GADF, as shown in Equation (3):

(3) $$\left\{\begin{array}{l} {\mathrm{GASF}}_{s}={\left[\cos(\theta_{s,i}+\theta_{s,j})\right]}_{n\times n}\\ {\mathrm{GADF}}_{s}={\left[\sin(\theta_{s,i}+\theta_{s,j})\right]}_{n\times n} \end{array}\right.$$

GASF encodes the cosine of angular sums, preserving temporal correlations and overall trends, and GADF encodes the sine of angular differences, excelling at capturing local variations and anomaly patterns [34]. To integrate these complementary representations, the GASF and GADF maps from each sensor are first summed element-wise and then concatenated, forming a 3D feature map of dimensions *S* × *n* × *n*:

(4) $$\mathrm{GAF}=\mathrm{Concat}\left[{\mathrm{GASF}}_{1}+{\mathrm{GADF}}_{1},\cdots ,{\mathrm{GASF}}_{S}+{\mathrm{GADF}}_{s}\right]$$

Specifically, GASF leverages cosine properties to reflect coordinated variation between similar time-point values, while GADF utilizes sine properties to highlight local fluctuation patterns through sensitivity to change rates. Element-wise fusion of GASF and GADF images integrates their complementary angular relationships, combining both representations into a unified input. This enables the CNN to simultaneously capture global trends and local details from the time series, enriching feature representation for subsequent learning stages without introducing computational overhead. The resulting fused representation provides a more informative feature space, thereby enhancing overall classification performance.

#### 3.1.2. CNN and MLP

The convolutional neural network is designed to automatically and efficiently extract both linear and nonlinear features from the GAF fused feature maps. The MLP then aggregates these features and generates the final prediction.

The CNN’s core consists of three convolutional blocks, each following an identical sequence of operations: a 3 × 3 convolution, batch normalization, a ReLU activation function, and a 2 × 2 max-pooling layer. Finally, a Dropout strategy with a probability of 0.1 is applied for mild regularization. Thus, the output of the convolutional layer at the *l*-th layer, denoted as $x_{l}$, can be described as

(5) $$x_{l}=\max\left(\mathrm{Relu}\left(\mathrm{BatchNorm}\left(\sum_{i\in I}k_{i}⊗x_{l-1}+b_{i}\right)\right)\right)$$

where $k_{i}$ denotes the convolution kernel, *x*_*l*−__1_ is the output feature map from the previous layer, $b_{i}$ represents the bias term, and *l* = 1, 2, 3.

The output of the final convolutional layer is flattened into a one-dimensional feature vector, denoted as $e_{f}$, which is then fed into the MLP. The MLP consists of two fully connected (FC) layers and an output layer. The output of the MLP, $e_{fc2}$, is computed by

(6) $$e_{fc2}=\mathrm{ReLU}\left(\left(\mathrm{ReLU}\left(e_{f}*w_{fc1}+b_{fc1}\right)\right)*w_{fc2}+b_{fc2}\right)$$

For single-label classification, $e_{fc2}$ is passed through a Softmax output layer to produce a normalized class probability distribution (i.e., ${\mathrm{output}}_{s}=\mathrm{softmax}(e_{fc2}*w_{o1}+b_{o1})$), and the class with the highest probability is the final prediction. For multi-label classification, a Sigmoid output layer generates independent confidence scores in [0, 1] for each class (i.e., ${\mathrm{output}}_{m}=\mathrm{sigmoid}(e_{fc2}*w_{o2}+b_{o2})$). A sample is assigned to a class if its corresponding score exceeds the decision threshold of 0.5.

The weights of the corresponding layers are denoted as $w_{fc1},w_{fc2},w_{o1}$ and $w_{o2}$; the biases are denoted as $b_{fc1},b_{fc2},b_{o1}$, and $b_{o2}$.

### 3.2. Gas Concentration Predictor

**Figure 7:**
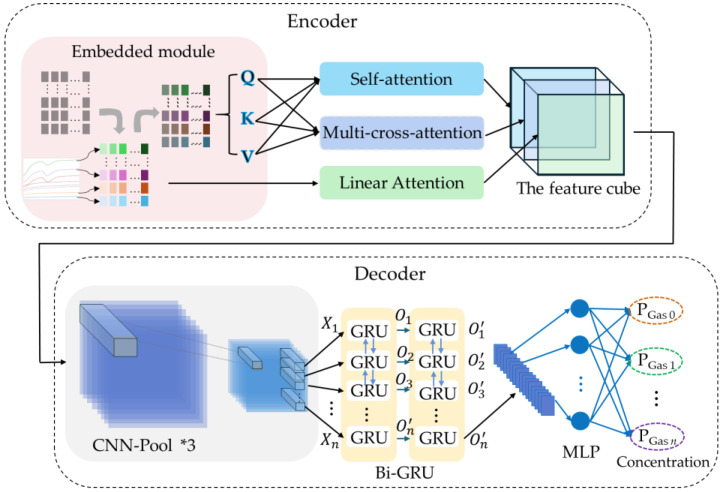
Gas concentration predictor framework.

To enhance the accuracy of gas concentration prediction in complex environments, this study proposes a gas concentration predictor that integrates multi-modal attention fusion with spatio-temporal decoupled learning. As illustrated in Figure 7, the model architecture consists of two modules: (1) an encoder that incorporates three heterogeneous attention mechanisms to extract multi-dimensional enhanced features from raw input sequences; and (2) a decoder that combines a CNN with a bidirectional GRU to capture spatial interaction patterns and bidirectional temporal dependencies, respectively.

Due to the parallel computing nature of attention mechanisms, positional embedding must be applied to the input data prior to the designed triple-branch attention processing. Then, three parallel attention branches are constructed: a single-head self-attention branch captures globally contextualized temporal dependencies; a multi-head self-attention branch extracts complex interactions from multiple subspaces; and a linear attention branch, which operates directly on the raw data, enhances numerical stability while mitigating information loss. The outputs of these three branches are concatenated to form a unified multi-representational feature cube, enabling comprehensive analysis by the decoder. Subsequently, this feature cube is processed by a CNN to extract inter-sensor spatial features, which are then flattened into temporal sequences and fed into a Bi-GRU to model deep bidirectional dependencies. Finally, a linear layer maps the abstract features to the concentration values of different gases. By integrating attention mechanisms, CNN, and RNN into a hybrid architecture, the model effectively achieves multi-modal feature fusion and decoupled learning in spatial and temporal dimensions. It simultaneously captures long- and short-term dependencies, spatial correlations, and dynamic temporal evolution, making it particularly suitable for high-precision concentration prediction in low-concentration and mixed-gas scenarios.

#### 3.2.1. Encoder Based on Multi-Branch Attention Fusion

Prior to attention processing, a sinusoidal–cosine embedding module injects positional information into the input sequence to preserve temporal ordering. For a sensor response $X=[X_{1},X_{2},\ldots ,X_{S}]\in {\mathbb{R}}^{n\times S}$ with sequence length *n* and feature dimension *S*, a positional encoding matrix $P\in {\mathbb{R}}^{n\times S}$ is generated and added to the input $\tilde{X}=X+P$. The encoding for position *i* and dimension *j* of *P* is computed by $P_{i,2j}=\sin\left(i/\left(10000^{2j/S}\right)\right)$ and $P_{i,2j+1}=\cos\left(i/\left(10000^{2j/S}\right)\right)$, where $i\in \left\{1,2,\cdots ,n\right\}$ and $j\in \left\{0,1,\cdots ,S/2-1\right\}$. This encoding formulation is chosen because it constructs a unified encoding space capable of expressing relative position information through linear transformations.

The encoded input $\tilde{X}$ is projected into Query (*Q*), Key (*K*), and Value (*V*) matrices for parallel processing. The single-head self-attention branch computes the scaled dot-product attention:

(7) $${\mathrm{Attention}}_{self}\left(Q,K,V\right)=\mathrm{softmax}\left(QK^{T}/\sqrt{d_{k}}\right)V$$

where $Q=\tilde{X}W^{Q}$, $K=\tilde{X}W^{K}$, $V=\tilde{X}W^{V}$, and the scaling by $\sqrt{d_{k}}$ prevents extremely small gradients. This mechanism captures global temporal dependencies across the sensor sequence, providing contextualized representations of the gas response dynamics.

To further enhance the model’s ability to capture diverse dependency patterns, the multi-head attention (MHA) mechanism is introduced. MHA projects the input into *h* distinct representation subspaces. For each head *i*, $Q_{i}=\tilde{X}W_{i}^{q}$, $K_{i}=\tilde{X}W_{i}^{k}$, $V_{i}=\tilde{X}W_{i}^{v}$, and ${\mathrm{Head}}_{i}=$$\mathrm{Attention}\left(Q_{i},K_{i},V\right)=\mathrm{softmax}\left(\left(Q_{i}K_{i}^{T}\right)/\sqrt{d_{k}}\right)V_{i}$. The outputs of all heads are concatenated and linearly projected to integrate the multi-subspace features:

(8) $${\mathrm{Attention}}_{MultiHead}\left(Q,K,V\right)=\mathrm{Concat}\left[{\mathrm{Head}}_{1},{\mathrm{Head}}_{2},\cdots ,{\mathrm{Head}}_{h}\right]W_{o}$$

To mitigate potential gradient vanishing or explosion issues during training, a linear attention module is incorporated to extract temporal features from the original data:

(9) $${\mathrm{Attention}}_{linear}=W_{l}*X+b_{l}$$

In the attention mechanism described above, the projection matrices, $W^{Q}$, $W^{K}$, $W^{V}$, $W_{i}^{q}$, $W_{i}^{k}$, $W_{i}^{v}$, $W_{O}$, $W_{l}$, and $b_{l}$, are all learnable parameters. The outputs from the three branches are concatenated to form a unified feature tensor *En*:

(10) $$En=\mathrm{Concat}\left[{\mathrm{Attention}}_{self},{\mathrm{Attention}}_{MultiHead},{\mathrm{Attention}}_{linear}\right]$$

This unified tensor provides the decoder with a rich, multi-perspective feature set for comprehensive analysis.

#### 3.2.2. Decoder Based on CNN, Bi-GRU and MLP

(1) CNN

The input to the CNN is the feature cube generated by the attention mechanism, while the convolutional block architecture (200 → 32 → 64 → 128 channels) remains identical to that employed in the classifier. The feature tensor produced by the CNN is subsequently flattened along the channel dimension to form a one-dimensional feature vector, which is then fed into a Bi-GRU for temporal modeling.

(2) Bi-GRU and MLP

**Figure 8:**
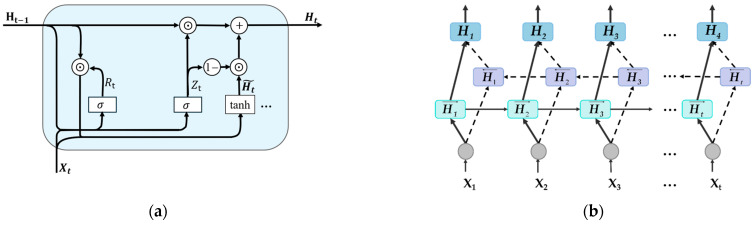
Schematic diagram of (**a**) GRU and (**b**) Bi-GRU.

In this study, the Bi-GRU operates on spatially enhanced feature sequences extracted by the CNN backbone, enabling joint spatio-temporal modeling and improving the discrimination of complex gas response patterns. The GRU cell, illustrated in Figure 8a, updates its hidden state through a gated mechanism:

(11) $$\left\{\begin{array}{l} R_{t}=\sigma \left(X_{t}W_{xr}+H_{t-1}W_{hr}+b_{r}\right)\\ Z_{t}=\sigma \left(X_{t}W_{xz}+H_{t-1}W_{hz}+b_{z}\right)\\ \tilde{H_{t}}=\tanh\left(X_{t}W_{xh}+(R_{t}⊙H_{t-1})W_{hh}+b_{h}\right)\\ H_{t}=Z_{t}⊙H_{t-1}+\left(1-Z_{t}\right){\tilde{H}}_{t} \end{array}\right.$$

where *R_t_* and $Z_{t}$ denote the reset gate and the update gate, respectively; $W_{xr}$, $W_{hr}$, $W_{xz}$, $W_{hz}$, $W_{xh}$, and $W_{hh}$ are all weight parameters; $b_{r}$, $b_{z}$, and $b_{h}$ are bias parameters; $X_{t}$ is the input at the current time step for both gates; $\tilde{H_{t}}$ is the candidate hidden state; $H_{t}$ and $H_{t-1}$ are the hidden state at the current and previous time step, respectively; and *σ* is the sigmoid function.

To leverage the complete data segment, we employ a Bi-GRU network that captures bidirectional temporal dependencies, yielding richer sequence representations. As shown in Figure 8b, it consists of two GRU layers that process the input sequence in opposite directions: a forward GRU that propagates information from *t* = 1 to *t* = t, and a backward GRU that propagates information from *t* = t to *t* = 1.

For an arbitrary time step t, the forward and backward hidden states are denoted as $\vec{H_{t}}$ and $\overset{\leftarrow }{H_{t}}$, respectively, and are updated as $\vec{H_{t}}={\mathrm{GRU}}_{f}\left(X_{t},\;\vec{H_{t-1}}\right)$ and $\overset{\leftarrow }{H_{t}}={\mathrm{GRU}}_{b}\left(X_{t},\;\overset{\leftarrow }{H_{t+1}}\right)$ following the update mechanism of the GRU unit. The hidden representations from both directions are then concatenated to obtain the final hidden state:

(12) $$H_{t}=\left[\vec{H_{t}};\;\overset{\leftarrow }{H_{t}}\right]$$

This representation integrates contextual information from past and future time steps, providing a more comprehensive feature representation for subsequent prediction tasks.

The decoder incorporates a five-layer fully connected MLP as its output module. The first four layers use the ReLU activation function to model nonlinear relationships between the extracted features and gas concentrations, whereas the output layer is linear, with its number of neurons equal to the number of target gas species. Each neuron in the output layer represents the predicted concentration of a specific gas.

### 3.3. Parameter Settings

The datasets were partitioned according to their characteristics and task objectives. The mixed-gas dataset (Dataset I) and the low-concentration dataset (Dataset II), which are used solely for regression tasks, were divided into training and test sets with a ratio of 8:2. The single-label classification and prediction datasets (Dataset III and IV) involve both classification and concentration prediction tasks. Owing to the increased training complexity of these dual-task scenarios, these datasets were further split into training, validation, and test sets with a ratio of 8:1:1, where the validation set was used for model selection and early stopping to improve generalization performance.

The gas classifier consists of three convolutional blocks with 32, 64, and 128 channels, respectively. Each block is equipped with 2 × 2 max-pooling and batch normalization. A FC layer with 256 units is then applied, and the output dimension is set to the number of gas categories (3, 4, and 3 for Datasets I, III and IV, respectively). In the gas concentration predictor, outputs of the three attention branches are projected to 200 dimensions. The subsequent convolutional module contains three convolutional blocks with 32, 64, and 128 channels. A Bi-GRU with a hidden size of 65 is adopted for temporal feature modeling. The resulting features are transformed by a four-layer FC network with dimensions of 198, 355, 168, and 43. Output layer predicts gas concentrations, with output dimensions of 3, 1, 1, and 1 for Datasets I–IV, respectively.

Both models are trained using the Adamax optimizer, with cross-entropy loss for the classification task and mean absolute error (MAE) for the regression task. The learning rate is consistently set to 0.001.

### 3.4. Evaluation Metrics

#### 3.4.1. Evaluation Metrics for Classification Tasks

For the classification task, the accuracy, precision, recall, and F1-score are selected as evaluation metrics. Given that each sample may belong to multiple classes simultaneously in multi-label classification, each class *C_j_* is treated as an independent binary classification problem. The formulas for these metrics are defined as follows.

Accuracy: In multi-label settings, exact match accuracy is commonly used. It is defined as the proportion of samples for which all labels are correctly predicted:

(13) $$\mathrm{Acc}=\frac{1}{N}\sum_{s=1}^{N}I(\hat{y_{s}}=y_{s})$$

where *N* is the total number of samples; $\hat{y_{s}}$ and $y_{s}$ denote the predicted and true labels of the *i*-th sample, respectively; and *I*(⋅) is the indicator function (equal to 1 if the condition is true, and 0 otherwise).

Precision: Precision for class $C_{j}$ is the proportion of true positives among all samples predicted as positive:

(14) $${\mathrm{Precision}}_{j}=\frac{TP_{j}}{TP_{j}+FP_{j}}$$

Recall: Recall for class $C_{j}$ is the proportion of true positives among all actual positives:

(15) $${\mathrm{Recall}}_{j}=\frac{TP_{j}}{TP_{j}+FN_{j}}$$

F1-score: F1-score for class $C_{j}$ is the harmonic mean of precision and recall:

(16) $$F1_{j}=2\times \frac{{\mathrm{Precision}}_{j}\times {\mathrm{Recall}}_{j}}{{\mathrm{Precision}}_{j}+{\mathrm{Recall}}_{j}}$$

In the above, $TP_{j}$ (true positives), $FP_{j}$ (false positives), and $FN_{j}$ (false negatives) denote the numbers of samples correctly predicted as class $C_{j}$, incorrectly predicted as class $C_{j}$, and belonging to class $C_{j}$ but not predicted as such, respectively. The overall precision, recall, and F1-score are then obtained by a weighted average over classes, with weights equal to the number of true samples in each class:

(17) $$\mathrm{Precision}=\sum_{j=1}^{C}\left(\frac{TP_{j}+FN_{j}}{\sum_{m=1}^{C}\left(TP_{m}+FN_{m}\right)}\times {\mathrm{Precision}}_{j}\right)$$

(18) $$\mathrm{Recall}=\sum_{j=1}^{C}\left(\frac{TP_{j}+FN_{j}}{\sum_{m=1}^{C}\left(TP_{m}+FN_{m}\right)}\times {\mathrm{Recall}}_{j}\right)$$

(19) $$F1=\sum_{j=1}^{C}\left(\frac{TP_{j}+FN_{j}}{\sum_{m=1}^{C}\left(TP_{m}+FN_{m}\right)}\times F1_{j}\right)$$

where *C* denotes the total number of classes.

#### 3.4.2. Evaluation Metrics for Regression Tasks

The regression performance is evaluated using the coefficient of determination (R^2^), root mean square error (RMSE), and mean absolute error (MAE). Higher R^2^ and lower RMSE and MAE values indicate better predictive performance. The overall R^2^, RMSE, and MAE for the *C* gas species are obtained by averaging the corresponding metrics calculated for each gas. The formulas are defined as follows:

(20) $$R^{2}=\frac{1}{C}\sum_{j=1}^{C}\left(1-\frac{\sum_{i=1}^{s}{\left(y_{i,j}-{\hat{y}}_{i,j}\right)}^{2}}{\sum_{i=1}^{s}{\left(y_{i,j}-{\bar{y}}_{j}\right)}^{2}}\right)$$

(21) $$\mathrm{MAE}=\frac{1}{C}\sum_{j=1}^{C}\left(\frac{1}{s}\sum_{i=1}^{s}\left|y_{i,j}-{\hat{y}}_{i,j}\right|\right)$$

(22) $$\mathrm{RMSE}=\frac{1}{C}\sum_{j=1}^{C}\sqrt{\frac{1}{s}\sum_{i=1}^{s}{\left(y_{i,j}-{\hat{y}}_{i,j}\right)}^{2}}$$

where *s* denotes the number of samples; $y_{i,j}$ and ${\hat{y}}_{i,j}$ are the true and predicted label values for the *j*-th gas in the *i*-th sample, respectively; and ${\bar{y}}_{j}$ is the mean of the true values for gas *j*.

## 4. Results and Analysis

In this section, extensive experiments were conducted on the four gas datasets to evaluate the proposed method. The experiments were performed on a workstation running Windows 10, equipped with an Intel Core i7-13700 processor, 64 GB of RAM, and an NVIDIA GeForce RTX 4080 GPU. The code was executed using Python 3.11.4 in Jupyter Notebook 6.5.4 within an Anaconda 3 environment.

### 4.1. Dataset I

**Figure 9:**
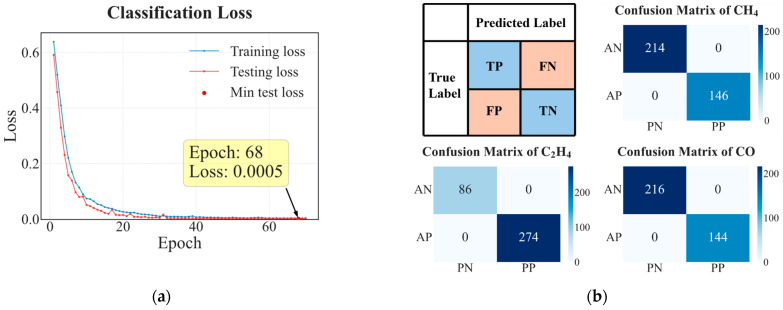
Classification performance on gas classification for Dataset I. (**a**) Training and testing loss over epochs; (**b**) confusion matrix for single-class classification of CO, CH_4_ and C_2_H_4_ on the test set.

Dataset I was used to evaluate the model’s performance in gas classification and concentration prediction. Figure 9 presents the loss trends over training epochs and the confusion matrices for the single-class classification of C_2_H_4_, CO, and CH_4_ on the test set. As shown in Figure 9a, the model converged rapidly within the first 30 epochs and continued to decrease steadily thereafter. The test loss reached its minimum value of 0.0005 at the 68th epoch and subsequently remained stable. The close alignment between training and testing losses throughout training indicates strong generalization with no evident overfitting. This stability can be traced to the GAF fusion strategy, which transforms one-dimensional sensor dynamics into structured two-dimensional representations, reducing the model’s tendency to fit spurious noise.

In the multi-gas classification task, the model may assign multiple gas labels to a single sample during prediction, which essentially corresponds to a multi-label classification problem. Since a conventional confusion matrix assumes that each sample belongs to only one class, it cannot accurately reflect class-wise performance in this scenario. Therefore, we adopt a one-vs-rest strategy, in which a separate confusion matrix is constructed for each gas category. Specifically, for the *i*-th gas, it is treated as the positive class, while all other gases are considered negative classes. Based on the correspondence between predicted labels and ground truth, the four outcomes (true positive, false positive, true negative, and false negative) are obtained to form the confusion matrix for that gas.

The confusion matrices (Figure 9b) demonstrate that all samples from the three gas classes (CO, CH_4_, and C_2_H_4_) were correctly classified, achieving 100% classification accuracy. This result indicates that the proposed multi-label classification framework with a sigmoid output layer is fully capable of distinguishing different gas species in complex mixed environments.

**Figure 10:**
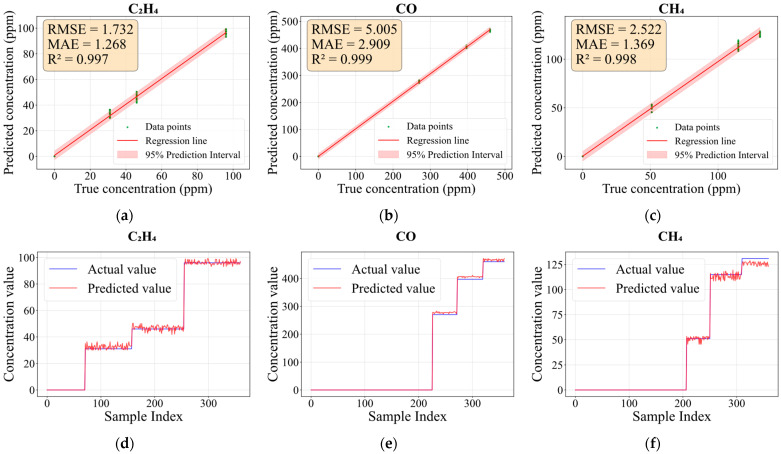
Comparison of predicted and actual concentrations of C_2_H_4_, CO and CH_4_ on the test set: (**a**–**c**) regression fitting with 95% prediction intervals; (**d**–**f**) step-change plots of concentrations.

The concentration prediction performance for C_2_H_4_, CO, and CH_4_ in Dataset I is evaluated in Figure 10. Figure 10a–c display the linear regression fitting between predicted and actual concentrations for each gas, along with error metrics (RMSE, MAE, and R^2^). Green dots represent samples, and the red area denotes the 95% prediction interval. Figure 10d–f show the step-change plots of predicted versus actual concentrations. As shown in the figures, the coefficients of determination (R^2^) for all three target gases exceed 0.997, indicating an excellent fit. The RMSE and MAE are low relative to the concentration range of each gas. Notably, CO exhibits higher RMSE and MAE values than C_2_H_4_ and CH_4_, which can be attributed to its wider concentration range. It is worth noting that the data points cluster tightly around the regression lines with narrow 95% prediction intervals. This demonstrates that the model accurately fits the actual concentration values across a broad range of concentrations. The step-change plots further reveal that the model can track concentration fluctuations of each component in real time. Moreover, when a particular gas component is absent, the predicted value remains near zero, confirming that the model possesses dual capabilities: gas identification and high-precision quantification.

We binarized the predicted concentration value of each output neuron: if the predicted value is greater than 0, it is recorded as 1, indicating the presence of that gas species; otherwise, it is recorded as 0. The binarized prediction labels were then compared with the true labels of the test set, and the model performance was evaluated using precision, recall, F1-score, and support. The results are presented in Table 5. As can be seen from the table, the proposed prediction model also exhibits effective gas identification capability.

This best performance can be attributed to two factors. First, each output neuron in the prediction model is tied to a specific gas species, enabling clear multi-label mapping. Second, the A–CNN–GRU architecture effectively captures inter-sensor dependencies through attention mechanisms, extracts discriminative features via convolutional layers, and models channel-wise dependencies using the Bi-GRU module.

### 4.2. Dataset II

**Figure 11:**
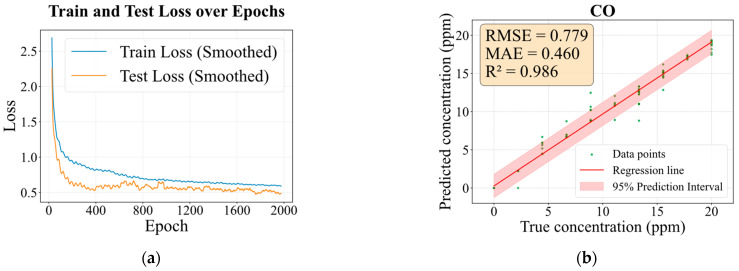
Evaluation of concentration prediction performance on Dataset II. (**a**) Regression loss function variation over epochs, (**b**) fitting curve for predicted versus actual concentrations and error metrics.

To evaluate the model’s predictive performance under low-concentration conditions, regression testing was performed on Dataset II, which contains only a single gas at low concentrations. Figure 11 presents the results, including the MAE loss over epochs and the predicted versus actual concentration fitting curve with error metrics. The rapid and stable convergence in Figure 11a, with closely matched training and test losses, stems from the MAF encoder and CNN–Bi-GRU decoder jointly enforcing learning of the physical gas response dynamics under weak-signal conditions. Consequently, the model achieves highly accurate predictions for CO within the 0–20 ppm range, with an RMSE of 0.779, an MAE of 0.460, and an R^2^ of 0.986.

Compared to the higher-concentration Dataset I, the model exhibits a slight decline in the coefficient of determination (R^2^) on the low-concentration Dataset II, with visible deviations between predicted and actual values for certain samples. This performance degradation stems primarily from the inherently weak electrical signals generated by gas sensors at low concentrations and is further exacerbated by pronounced relative humidity fluctuations (15–75% RH), under which these weak signals are readily obscured by noise. Nevertheless, the model sustains high predictive accuracy under such adverse conditions. This robustness is largely attributed to the integrated three-branch attention mechanism, which adaptively enhances the raw time-series data through dynamic weighting. By suppressing humidity-induced interference and amplifying subtle yet informative gas response features, the mechanism ensures reliable performance. These findings confirm that the proposed model retains strong deep feature extraction capability and stable quantitative prediction even in extreme, low-concentration, and high-interference environments.

### 4.3. Dataset III

To mitigate the cross-category prediction bias caused by heterogeneous gas concentration distributions, an identification–prediction cascade architecture is proposed. The framework constructs a gas-specific expert system in which each gas category is associated with an independent regression sub-network. During training, the classification and regression modules are optimized separately. Unlike conventional approaches, the regression module incorporates a category-aware masking mechanism that routes samples of the same gas category to the corresponding regressor.

**Figure 12:**
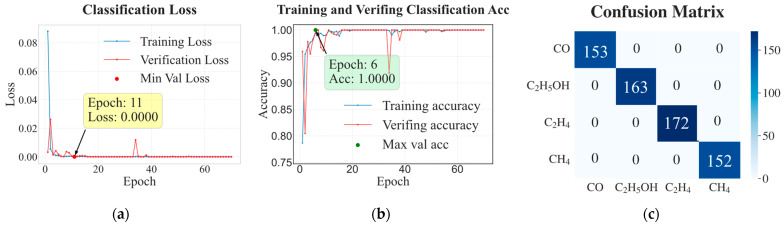
Classification performance on gas classification for Dataset III. (**a**,**b**) Classification loss and accuracy on training and test sets during training; (**c**) confusion matrix for the four gas types.

Figure 12 presents the classification loss and accuracy on the training and test sets over training epochs, together with the confusion matrix for the four gas categories. As shown in Figure 12a,b, both training and validation losses decrease rapidly, with the validation loss dropping to 0.00 by Epoch 11 and converging to near-zero values after several epochs, while the corresponding accuracies quickly reach 1.00 by Epoch 6 and remain stable throughout the subsequent training process. This behavior indicates that the classifier effectively captures the discriminative patterns of the dataset without overfitting. The confusion matrix in Figure 12c further confirms the classification performance. No misclassification is observed among the four gas categories (CO, C_2_H_5_OH, C_2_H_4_, and CH_4_), with all samples located on the diagonal elements of the matrix. This perfect classification result provides a reliable foundation for the subsequent category-specific routing in the regression stage.

**Figure 13:**
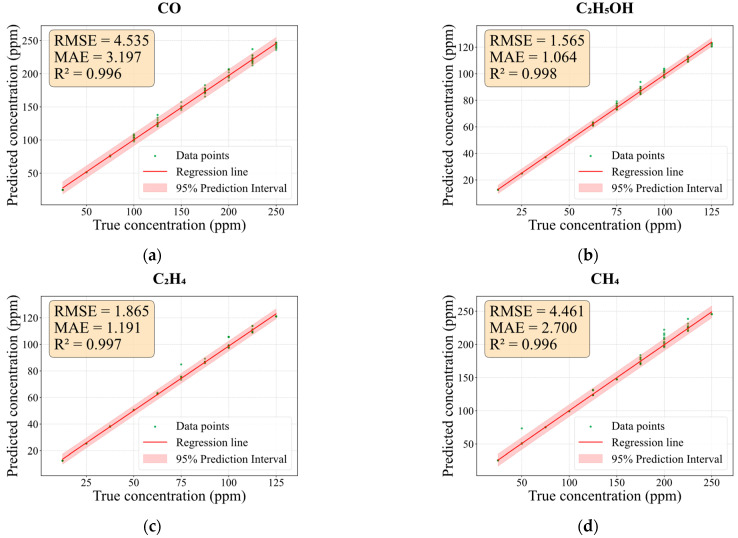
Linear regression analysis and error metrics for concentration prediction on Dataset III for (**a**) CO; (**b**) C_2_H_5_OH; (**c**) C_2_H_4_; and (**d**) CH_4_.

Based on this architecture, the regression networks demonstrate excellent concentration prediction performance for all gases, as illustrated in Figure 13. The predicted concentrations exhibit strong linear agreement with the ground-truth values, yielding R^2^ values of 0.996, 0.998, 0.997, and 0.996 for CO, C_2_H_5_OH, C_2_H_4_, and CH_4_, respectively. Benefiting from this identification–prediction framework, all test samples were correctly classified and routed to their dedicated regression branches, thereby substantiating the necessity of the category-masking mechanism. Architecturally, this approach mitigates the performance decline seen in unified models processing heterogeneous gases, moving from broad generalization toward targeted precision.

### 4.4. Dataset IV

**Figure 14:**
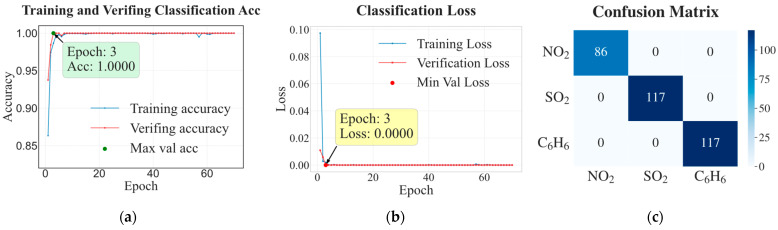

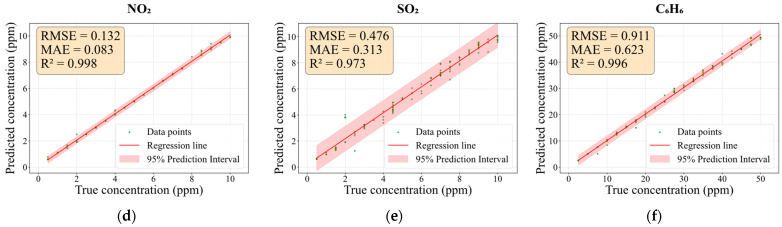
Classification and concentration prediction performance for Dataset IV. (**a**) Comparison of classification accuracy curves for training and validation sets; (**b**) trend of classification loss with training epochs; (**c**) confusion matrix for NO_2_, SO_2_, and C_6_H_6_; (**d**–**f**) predicted concentration versus actual concentration fitting curves for NO_2_, SO_2_, and C_6_H_6_, respectively.

To further validate the effectiveness of the proposed expert architecture and avoid potential dataset-specific bias, the identification–prediction framework was additionally evaluated on a self-constructed laboratory dataset. It was recorded using a sensor array comprising commercial MOX sensors (WSP1110, WSP2110, and MQ136), demonstrating the framework’s transferability to independently designed hardware beyond the public benchmark datasets. As shown in Figure 14a,b, the model exhibited fast and stable convergence during training. Both training and validation losses decreased rapidly in the early epochs and reached near-zero values by the third epoch, while the corresponding accuracies simultaneously increased to 1.000 and remained stable thereafter. The close agreement between training and validation losses indicates that the model effectively captured discriminative patterns and maintained strong generalization capability.

The confusion matrix in Figure 14c further validates the classification performance. All samples of NO_2_, SO_2_, and C_6_H_6_ were correctly classified across multiple independent test sets, with all samples located on the diagonal elements of the matrix. This perfect classification accuracy provides a reliable basis for the subsequent concentration regression stage.

Regarding the concentration prediction task, the model demonstrated a strong linear correlation between predicted and actual concentrations, as illustrated in Figure 14d–f. The regression results achieved R^2^ values of 0.998, 0.973, and 0.996 for NO_2_, SO_2_, and C_6_H_6_, respectively, with corresponding RMSE values of 0.132, 0.476, and 0.911, and MAE values of 0.083, 0.313, and 0.623. The relatively broader prediction interval for SO_2_ can be attributed to the cross-sensitivity of the SO_2_ sensing unit, while the slightly larger errors for C_6_H_6_ are likely related to its wider concentration range. Nevertheless, the high R^2^ values indicate stable prediction performance across different concentration levels.

Consistent with the findings on Dataset III, these results indicate that the proposed identification–prediction framework alleviates the loss of model specificity arising from inconsistent data distributions in multi-class gas detection tasks. This further supports its practical viability in real-world deployment.

## 5. Discussion

### 5.1. Comparative Experiment

To evaluate the classification and prediction performance of the proposed model, 12 methods were considered for comparative experiments. The classic machine learning methods included support vector machine (SVM), k-nearest neighbors (KNN), extreme learning machine (ELM), and regression models (RM), while the recently reported methods comprised 2L-ARNN [37], PMH-TCN [38], A-GRU [39], 1D-CNN [40], 3D-CNN [41], and DWCNN-LSTM [24].

To enhance the reliability of the experimental results, Dataset I, which contains both gas classification and concentration prediction information, was selected for comparative evaluation. We applied a 5-fold cross-validation scheme to all models. Specifically, the preprocessed dataset was randomly partitioned into five equal-sized, mutually exclusive subsets. In each iteration, four subsets were used to train the model, while the remaining subset served as the independent test set. This process was repeated five times, ensuring that each data point was utilized for testing exactly once. For classical machine learning methods, the raw sensor time-series data were first normalized using Z-score to eliminate scale differences. Subsequently, five statistical features (maximum, minimum, mean, standard deviation, and variance) were extracted, transforming each sample into a 40-dimensional feature vector as model input. In contrast, deep learning methods directly utilized the preprocessed sensor response sequences during the response phase as input, thereby fully leveraging their end-to-end feature learning capability. To ensure a fair comparison, several adaptations were made to certain baseline models, including adjusting the number of output neurons according to the number of gas categories and modifying the output layer activation functions to support concentration prediction tasks. All other hyperparameters were kept consistent with those reported in the original studies, ensuring that each model operated under its near-optimal conditions.

Table 6 presents the classification and prediction performance of all compared methods. The reported inference time represents the median value over 1000 single-sample predictions. The following observations can be drawn from the results.

(1) In the gas classification task, KNN and SVC achieved accuracies of 81.67% and 91.11%, respectively, while ELM(C) only reached 47.78%. In the concentration prediction task, SVR and ELM(R) obtained overall R^2^ values of 0.9520 and 0.9593, respectively, accompanied by relatively high RMSE and MAE. These results indicate limited capability in modeling complex nonlinear relationships, attributed to the fact that traditional methods rely on handcrafted features and static mappings that are insufficient to capture the dynamic characteristics of gas sensor responses.

(2) As most deep learning models have achieved performance saturation in classification, the prediction task provides a more discriminative evaluation. Regarding regression performance, recurrent-based models such as 2L-ARNN, PMH-TCN and A-GRU achieved overall R^2^ values of 0.9582, 0.9699, and 0.9632, respectively, with RMSE values all exceeding 10. This may be due to their limited ability to extract critical features and their susceptibility to information attenuation over long sequences. In contrast, convolution-based models, including 1D-CNN and 3D-CNN, achieved higher overall R^2^ values of 0.9877 and 0.9942, respectively, with RMSE reduced to 7.4834 and 5.7612, and significantly lower MAE, indicating strong capability in local feature extraction. The hybrid DWCNN-LSTM model yielded an overall R^2^ of 0.9793, with RMSE and MAE values that compare favorably against several competing approaches. By comparison, the proposed model achieved the best performance across all evaluation metrics, with an overall R^2^ of 0.9897 and R^2^ values of 0.9720 (C_2_H_4_), 0.9979 (CO), and 0.9897 (CH_4_). In addition, it attained the lowest RMSE and MAE, indicating superior prediction accuracy, more concentrated error distribution, and improved stability. This superior performance is attributed to its effective integration of multi-scale feature extraction with temporal dynamic modeling, thereby capturing the complex relationships in gas response processes.

(3) Although conventional methods and highly efficient convolutional models such as 3D-CNN achieve extremely fast inference (0.017 ms), the proposed model attains an inference time of 2.8885 ms, which remains well within the millisecond range. The end-to-end latency of the proposed method is quantified as $T_{\mathrm{total}}=T_{\mathrm{response}}+T_{\mathrm{detection}}+T_{\mathrm{inference}}$, where $T_{\mathrm{response}}$ (60–180 s) is the inherent physical sensor rising time, $T_{\mathrm{detection}}$ (0.2–0.5 s) is the steady-state confirmation window, and $T_{\mathrm{inference}}$ (2.89 ms) is the model execution time. As the algorithmic delay (<3 ms) is negligible compared to the physical response, the total latency is governed by sensor kinetics rather than computational complexity, achieving >50% reduction in measurement cycle versus traditional methods (300–600 s) by eliminating the prolonged steady-state holding and recovery phases.

### 5.2. Ablation Experiment

**Figure 15:**
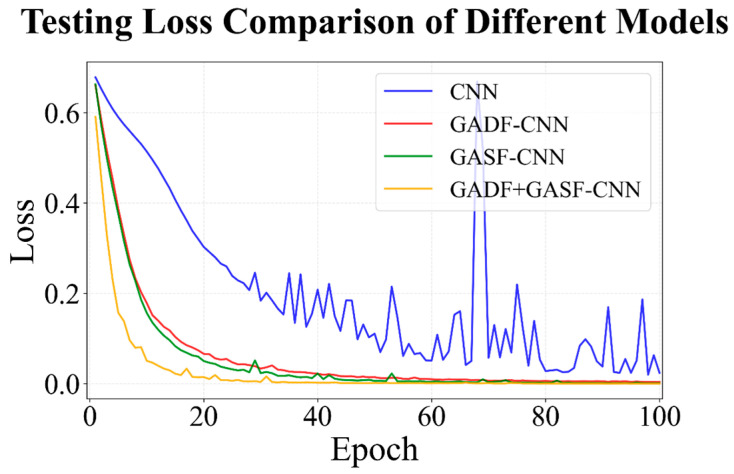
Testing loss of different classification models over epochs on Dataset I.

To validate the effectiveness of the proposed model combination strategy, systematic ablation experiments were conducted for both the classification and prediction models, with results presented in Figure 15 and Table 7.

For the classification model, the performance of the original CNN, GADF-CNN, GASF-CNN, and the fused model (GASF+GADF-CNN) was systematically compared. Compared with the conventional CNN operating directly on raw time-series data, the introduction of GADF or GASF transformations led to faster convergence during early training stages and a more pronounced reduction in loss. This indicates that GAF-based representations enhance the structural information of the input, thereby improving feature discriminability and accelerating the optimization process. Furthermore, the fused model integrating both GADF and GASF achieved the fastest convergence throughout training, stabilizing after approximately 30 epochs. In terms of convergence stability, the original CNN exhibits noticeable fluctuations during the middle and late training stages, including occasional spikes in loss, suggesting an unstable optimization process. In contrast, both GADF-CNN and GASF-CNN show smoother loss curves, while the fused model demonstrates nearly fluctuation-free behavior. This confirms that feature fusion effectively enhances model robustness.

To further evaluate the contribution of each key module in the proposed prediction framework, ablation experiments were conducted on the challenging low-concentration Dataset II, including A-CNN, CNN-GRU, and the hybrid A-CNN-GRU model. The results are summarized in Table 7. All individual models achieve relatively high prediction accuracy but exhibit trade-offs across different evaluation metrics. The A-CNN model attains the lowest MAE (0.4225), indicating superior performance in controlling average error; however, its lower R^2^ (0.9618) suggests limited capability in capturing the overall data distribution. By incorporating temporal modeling, the CNN-GRU model improves R^2^ to 0.9771, demonstrating enhanced ability to characterize the dynamic response of gas signals. Nevertheless, its RMSE and MAE remain slightly higher than the optimal values, reflecting residual prediction variability.

In contrast, the proposed A-CNN-GRU model achieves the best overall performance, with RMSE significantly reduced to 0.7794 and R^2^ elevated to 0.9860, indicating a superior balance between fitting accuracy and error control. Although its MAE is marginally higher than that of A-CNN, the overall error is lower and more stable, demonstrating enhanced generalization capability. This improvement can be attributed to the effective integration of convolutional feature extraction and GRU-based temporal modeling, enabling comprehensive capture of complex dynamic patterns in gas sensor responses. Experimental results confirm that the introduced modules in the proposed framework are complementary rather than redundant. Their integration enhances performance across varying task complexities and environmental conditions, providing a robust foundation for gas identification and concentration prediction in electronic nose systems.

### 5.3. The Impact of Data Length on Model Performance

To investigate the minimum data requirements for reliable gas identification and quantification, we evaluated the impact of input sequence length on model performance using Dataset I. The complete rising segment (spanning from exposure onset through the rising transient to the initial steady-state establishment) was divided into four equal intervals, yielding experimental groups utilizing 1/4, 2/4, 3/4, and the full (4/4) of this rising segment as model inputs. All preprocessing parameters and model architectures remained consistent with Section 2.2 and Section 4.1.

**Figure 16:**
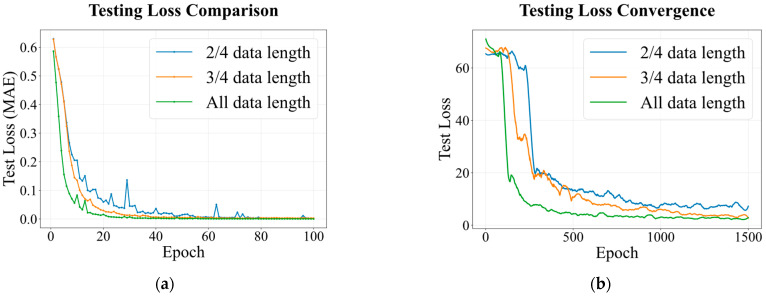
Variation in test loss with epoch under different data lengths (**a**) gas classifier (**b**) concentration predictor.

Figure 16a illustrates the variation in test loss with training epochs for the gas classifier under different input lengths. When utilizing only the initial 1/4 of the rising segment, the model fails to achieve numerical convergence due to a severe lack of discriminative features, resulting in unstable loss values throughout training. For the 2/4 and 3/4 length segments, while eventual convergence is achieved, the onset of convergence is delayed by approximately 20 epochs compared to the full-length identification model, accompanied by significant loss fluctuations during the training process. The prediction model utilizing the complete rising segment exhibits rapid convergence within the first 200 epochs and maintains stable, low-loss values thereafter. This indicates that pushing too hard to minimize data length comes at the cost of fitting robustness.

The concentration prediction performance under varying input lengths is presented in Figure 16b and quantified in Table 8. As the input length decreases, prediction accuracy deteriorates across all gas species. Taking CO as an example, the RMSE at 2/4 length (27.22) is 9.4 times that of the full length (2.90). The sensitivity to sampling length varies across different gas species. For instance, while CH_4_ retains a certain degree of predictive capability at 2/4 length (R^2^ = 0.9618), the performance for C_2_H_4_ is markedly inferior (R^2^ = 0.7665). This disparity reflects the diverse adsorption kinetic characteristics of different gas molecules; premature truncation of sampling creates blind spots for gases with slower response rates. The experiments confirm that utilizing the complete rising data segment is the effective length required to ensure the successful completion of identification and prediction tasks in complex gas mixture scenarios.

## 6. Conclusions

This study proposes a dual-model framework for rapid gas identification and concentration estimation in electronic nose systems. A composite Gramian Angular Field representation fusing GASF and GADF is introduced to transform time-series sensor data into discriminative two-dimensional feature maps, enabling end-to-end gas classification via a CNN-based classifier. For concentration prediction, an attention-augmented CNN–Bi-GRU architecture is developed to capture multi-scale spatial features and bidirectional temporal dependencies. Furthermore, a cascaded identification–prediction scheme is proposed to mitigate cross-category prediction bias arising from heterogeneous data distributions in single-gas scenarios. A key advantage of this framework is that it obviates the need for complete sensor response cycles, enabling rapid and accurate identification and quantification by leveraging the rising phase from initial exposure to early steady state. The effectiveness of the proposed method is validated on three publicly available datasets and a dataset collected in our lab. Experimental results demonstrate that the classification model achieves 100% accuracy across all evaluation scenarios, while the concentration predictor exhibits excellent quantitative reliability across diverse gases and concentration ranges.

In future work, we will extend the framework to complex multi-component mixtures beyond binary combinations, further reduce sensing latency through early transient feature analysis and lightweight architecture design, and advance embedded system deployment and real-world testing under practical operating constraints.

## Figures and Tables

**Table 1:** Gas concentration values (ppm) for the four levels in Dataset I.

Gas	Zero	Low	Medium	High
C_2_H_4_	0	31	46	96
CO	0	270	397	460
CH_4_	0	51	115	131

**Table 2:** The concentration levels and class labels for various gases in Dataset III.

Analyte	Label	Concentration (ppm)
CO	[0]	25.0, 50.0, 75.0, 100.0, 125.0, 150.0, 175.0 200.0, 225.0, 250.0
C_2_H_5_OH	[1]	12.5, 25.0, 37.5, 50.0, 62.5, 75.0, 87.5, 100.0, 112.5, 125.0
C_2_H_4_	[2]	12.5, 25.0, 37.5, 50.0, 62.5, 75.0, 87.5, 100.0, 112.5, 125.0
CH_4_	[3]	25.0, 50.0, 75.0, 100.0, 125.0, 150.0, 175.0 200.0, 225.0, 250.0

**Table 3:** Sample numbers, class labels, and concentration values for the three gases in Dataset IV.

Gas	Number	Label	Concentration (ppm)
NO_2_	80	[0]	0.5, 1.0, 1.5, 2.0, 2.5, 3.0, 3.5, 4.0, 4.5, 5.0, 5.5, 6.0, 6.5, 7.0, 7.5, 8.0, 8.5, 9.0, 9.5, 10.0
SO_2_	120	[1]	2.5, 5.0, 7.5, 10.0, 12.5, 15.0, 17.5, 20.0, 22.5, 25.0, 27.5, 30.0, 32.5, 35.0, 37.5, 40.0, 42.5, 45.0, 47.5, 50.0
C_6_H_6_	120	[2]	0.5, 1.0, 1.5, 2.0, 2.5, 3.0, 3.5, 4.0, 4.5, 5.0, 5.5, 6.0, 6.5, 7.0, 7.5, 8.0, 8.5, 9.0, 9.5, 10.0

**Table 4:** Multi-label coding scheme for gas types in Dataset I.

Types	Multi-Label		
	CH_4_	C_2_H_4_	CO
Air	0	0	0
CO	0	0	1
C_2_H_4_	0	1	0
CH_4_	1	0	0
C_2_H_4_-CH_4_	1	1	0
C_2_H_4_-CO	0	1	1

**Table 5:** Binary classification performance metrics of gas concentration based on prediction results from the test set.

Gas	Precision	Recall	F1-Score	Support
C_2_H_4_	1.0	1.0	1.0	289
CO	1.0	1.0	1.0	134
CH_4_	1.0	1.0	1.0	153

**Table 6:** Performance of the compared methods on Dataset I.

Method	Acc	F1-Score	R^2^ C_2_H_4_	R^2^ CO	R^2^ CH_4_	R^2^	RMSE	MAE	Time (ms)
KNN	0.8167	0.9337	/	/	/	/	/	/	4.5132
SVC	0.9111	0.9712	/	/	/	/	/	/	1.4283
ELM(C)	0.4778	0.7985	/	/	/	/	/	/	0.0074
SVR	/	/	0.8924	0.9774	0.9863	0.9520	15.2727	10.6416	0.3453
ELM(R)	/	/	0.9526	0.9514	0.9739	0.9593	9.1105	7.1099	0.0754
2L-ARNN [37]	1.0	1.0	0.9119	0.9829	0.9797	0.9582	13.4870	7.3107	0.6146
PMH-TCN [38]	/	/	0.9315	0.9906	0.9875	0.9699	10.3480	5.1809	3.4311
A-GRU [39]	1.0	1.0	0.9201	0.9851	0.9844	0.9632	12.9065	7.3486	0.5643
1D-DCNN [40]	1.0	1.0	0.9739	0.9958	0.9933	0.9877	7.4834	4.9114	0.3124
3D-CNN [41]	/	/	0.9883	0.9962	0.9982	0.9942	5.7612	3.1009	0.0170
DWCNN-LSTM [24]	1.0	1.0	0.9729	0.9673	0.9936	0.9793	6.9275	4.9695	0.5769
Our model	1.0	1.0	0.9720	0.9992	0.9979	0.9897	4.3507	1.7634	2.8885

**Table 7:** Performance metrics for gas concentration prediction in ablation experiments on Dataset II.

Method	RMSE	MAE	R^2^
A-CNN	1.2309	**0.4225**	0.9618
CNN-GRU	0.9436	0.4299	0.9771
A-CNN-GRU	**0.7794**	0.4596	**0.9860**

Bold values indicate the best performance metrics under the corresponding methods.

**Table 8:** Evaluation metrics of prediction performance on the test set for different data segments in Dataset I.

Gas	2/4 Data Length			3/4 Data Length			All Data Length		
	MAE	RMSE	R^2^	MAE	RMSE	R^2^	MAE	RMSE	R^2^
C_2_H_4_	7.6877	16.3643	0.7665	3.1032	7.2990	0.9542	1.7320	5.0050	**0.9970**
CO	8.4328	27.2221	0.9785	1.4822	3.3325	**0.9997**	1.2680	2.9090	0.9990
CH_4_	2.3455	9.9789	0.9618	2.2980	5.8927	0.9883	2.5220	1.3690	**0.9980**

Bold values indicate the best R^2^ for each class of gases with different input lengths.

## Data Availability

The original contributions presented in this study are included in the article. Further inquiries can be directed to the corresponding author(s).
